# Itching and Its Treatment

**Published:** 1909-06-05

**Authors:** 


### Dermatology.

### ITCHING AND ITS TREATMENT.

Itching, or pruritus, is a prominent symptom of many skin affections. It may also occur without any skin eruption, or such skin lesions as are present may be the result of scratching. Very often it is on account of the pruritus that a patient seeks advice, rather than because of the inconvenience of any eruption, or for other symptoms. But it is the duty of the medical man, not merely to attempt to treat this symptom, but to find out the cause of it, and, if possible, to remove it.

### Differential Diagnosis.

Always, in the presence of pruritus of whatever variety, and in whatever station of life the patient may be, it is essential first of all to exclude the parasitic affections, *scabies* and *pediculosis*. If the patient be young, one must suspect scabies; if elderly, pediculosis. Some indication of the presence of either of these affections will be given by the nature of the eruption and its distribution. An ill-defined scratched papular eruption involving more or less of the whole body below the neck suggests scabies. Scratched papules and scratch-marks on the skin of the back in an elderly person suggest pediculosis.

But to diagnose these complaints with certainty it is necessary to find the typical burrows in the case of scabies—about the wrists or fingers or sides of the feet, and on the penis in men—and the pediculus in pruritus of old people, in the folds of the linen about the neck.

When these parasitic affections have been definitely excluded, we must next examine the eruption to make out whether it is one or other of the more common eruptions which are accompanied by pruritus. We think of *eczema*, of *lichen planus*, of *urticaria*, and in children of *lichen urticatus*. If the eruption be eczema, we see the characteristic red, œdematous skin, cracked and oozing, or studded with pin-point erosions, vesicles, or crusts. If it be lichen planus, we have the violet-coloured, flat-topped, angular papules of this eruption, situated especially about the wrists and the inner sides of the knees. In urticaria we see the well-known wheals. In lichen urticatus, the papule with erythematous or urticarial halo. But it must not be forgotten that eczema may be the result of scabies, especially of scabies that has been over-treated with sulphur ointment; nor that, occasionally, scabies and lichen planus may be present together in the same patient; and that urticaria may accompany both scabies and pediculosis.

Having now excluded all of these affections—scabies, pediculosis, eczema, lichen planus, urticaria, lichen urticatus—we must next endeavour to find out whether the itching depends upon some general condition. We know that a common cause of pruritus is some passing *gastro-intestinal disturbance*, that pruritus is common in *diabetes*, in *Bright's disease*, and in *jaundice*. It may also occur in patients with *cardiac disease*, in *pregnant women*, and—a very important fact to remember—in patients suffering from *malignant disease of the stomach or liver*. All these points should be carefully investigated before attempting to prescribe for this symptom.

There are two other less common causes of pruritus which one has to bear in mind, but which can scarcely be diagnosed with certainty until the patient has been under observation for some time. These are *dermatitis herpetiformis* and the early stage of *mycosis fungoides*. *Prurigo of Hebra*, a common cause of life-long pruritus in Vienna among the poor, is seldom met with in this country.

### Treatment.

Having made the diagnosis of scabies or of pediculosis, the treatment is aimed at the cause of the disease, and the well-known applications for these complaints generally quickly remove this troublesome symptom. In scabies, however, a good deal of itching may be complained of even after the disease is cured. This is generally speedily removed by a mild tar lotion. The treatment of eczema need not be entered into here. In lichen planus and urticaria the same applications are useful as in pruritus from other causes. In pruritus dependent upon the disturbance of the digestive functions the bowels must be regulated and careful attention given to the diet. Alcohol, coffee, and any particular kind of food which has been found to disagree must be avoided. Appropriate treatment for diabetes and albuminuria will generally relieve the itching which occurs in these conditions. Cardiac cases find relief from small doses of digitalis.

There are certain empirical remedies which have been found useful in obstinate cases, or as temporary measures in all cases. These are: quinine in large doses; antipyrin, in doses of gr. v., gradually increased. Pilocarpin, gr. $\frac{1}{16}$ to $\frac{1}{10}$, by the mouth when the itching is most troublesome. The good effect of this remedy is often very striking. Cannabis indica is recommended in the pruritus of old people, in doses of 5 to 20 ♏. well diluted, three times a day, after food.

In all cases local applications are useful, and the list of such applications is a long one. Among the most useful are lotions containing liquor picis carbonis (ʒj. ad ʒx.), acidum carbolicum (♏xl. ad ℥x.), sanitas ʒss. ad ℥x.). Alkaline lotions: bicarbonate of soda or borax ʒij. to ℥x., with or without a few drops of dilute hydrocyanic acid; lotions containing a powder in suspension, oxide of zinc, calamine, talc. Or a powder may be suspended in the tar or alkaline lotion. Sometimes cold sponging with vinegar and water, or with alcohol and ether, followed by powdering with starch powder, gives relief. Lotions may be conveniently applied by means of a spray. A lotion of perchloride of mercury (gr. v. ad x. ad ℥x.) is cleanly and odourless. It is useful to bear in mind several prescriptions, for often one relieves where another fails.

